# Comparison of brain gray matter volume changes in peritoneal dialysis and hemodialysis patients with chronic kidney disease: a VBM study

**DOI:** 10.3389/fnins.2024.1394169

**Published:** 2024-04-26

**Authors:** Fenglin Xiao, Lili Zhou, Yan Li, Chaoyang Zhang, Ying Liu, Huan Yu, Xiaoping Li, Chunyu Wang, Xinxin Yin, Xin Gao

**Affiliations:** ^1^Department of Nephrology, First Medical Center of Chinese PLA General Hospital, Nephrology Institute of the Chinese People’s Liberation Army, National Key Laboratory of Kidney Diseases, National Clinical Research Center for Kidney Diseases, Beijing Key Laboratory of Kidney Disease Research, Beijing, China; ^2^7th Department of Health Cadre, The Second Medical Center of Chinese PLA General Hospital, Beijing, China; ^3^Department of Radiology, The 941th Hospital of the PLA Joint Logistic Support Force, Xining, China; ^4^Department of Nephrology, Liangxiang Hospital, Beijing, China; ^5^Shanghai Universal Medical Imaging Diagnostic Center, Shanghai, China

**Keywords:** chronic kidney disease, peritoneal dialysis, hemodialysis, voxel morphometry, cognitive impairment

## Abstract

**Objective:**

This study aims to compare gray matter volume changes in patients with chronic kidney disease (CKD) undergoing peritoneal dialysis (PD) and hemodialysis (HD) using voxel-based morphometry (VBM).

**Methods:**

A total of 27 PD patients, 25 HD patients, and 42 healthy controls were included. VBM analysis was performed, and cognitive function was assessed using the Mini-Mental State Examination (MMSE) and the Montreal Cognitive Assessment Scale (MoCA). The correlation between cognitive function and changes in brain gray matter volume was analyzed.

**Results:**

Both peritoneal dialysis and hemodialysis patients had partial gray matter volume reduction compared to the controls, but the affected brain regions were not uniform. The hemodialysis patients had greater volume reduction in certain brain regions than the PD patients. The MMSE and MoCA scores were positively correlated with gray matter volume changes.

**Conclusion:**

Different dialysis modalities cause damage to specific areas of the brain, which can be detected using VBM. VBM, combined with cognitive function assessment, can help detect structural brain changes and cognitive impairment in patients with different dialysis modalities. The comprehensive application of VBM in the field of neurological function deserves further exploration.

## 1 Introduction

Chronic kidney disease (CKD) is a chronic progressive disease caused by multiple factors with high morbidity and mortality, caused by such as diabetes and hypertension (Bikbov et al. 2020; Kalantar-Zadeh et al., 2021). Previous studies have shown that the global burden of CKD is enormous and growing. By 2040, CKD will become the fifth most deadly disease worldwide (Foreman et al., 2018). China accounts for approximately 19% of all CKD patients worldwide (2020), with more than 80 million CKD patients.

CKD can be classified into five stages according to the estimated glomerular filtration rate (eGFR). When patients progress to the end stage or CKD stage 5 (eGFR <15 mL/min/1.73 m^2^), renal replacement therapy, mainly including maintenance dialysis and kidney transplantation, is required (2020). Currently, the main clinical modalities of maintenance dialysis include hemodialysis and peritoneal dialysis. CKD often involves vascular pathology, such as hypertension and atherosclerosis, which can compromise blood flow to the brain, leading to vascular cognitive impairment. Some studies have reported a higher incidence of cognitive impairment in patients undergoing maintenance dialysis than in healthy controls (Hiramatsu et al., 2020; Gamage et al., 2022). Patients with CKD with significant neurological symptoms may receive more attention and appropriate treatment (Li et al., 2019a, 2021). However, doctors do not easily detect cognitive dysfunction in patients, and patients have decreased quality of life and poor prognosis (Hiramatsu et al., 2020).

Common methods of assessing cognitive function are the Mini-Mental State Examination (MMSE) and the Montreal cognitive assessment (MoCA) (Jia et al., 2021; Li et al., 2022). However, the onset of cognitive impairment is insidious, and its early stages cannot be accurately assessed using scales. Conventional magnetic resonance imaging (MRI) is highly sensitive to significant brain atrophy. However, it has limitations in the diagnosis of early brain atrophy and neurological abnormalities in end-stage renal disease (ESRD) patients with or without subtle vascular damage but with cognitive impairment. Voxel-based morphometry (VBM) is a whole-brain, non-invasive technique for characterizing regional brain volume and tissue structure differences in structural MRI (Zacková et al., 2021), which can assess microstructural changes in brain atrophy as well as early lesions. Recently, VBM has been reported to detect cortical atrophy and brain microstructural differences (Li et al., 2019b).

In our study, we propose to explore the effects of different dialysis modalities on structural brain volume in patients with CKD using the VBM method and further analyze the correlation between brain gray matter volume changes and traditional cognitive impairment scores.

## 2 Materials and methods

### 2.1 Participants and clinical data collection

We enrolled 52 patients with CKD who had ESRD and underwent dialysis in the Liangxiang Teaching Hospital of Capital Medical University from June 2021 to June 2022. Inclusion criteria were as follows: (1) patients underwent conventional hemodialysis 3 times per week for 3–4 h each time or peritoneal dialysis treatment 3–4 times per day for 4 h each time; (2) patients who were at least 18 years of age; and (3) patients underwent MRI imaging with head movements of less than 1.0 mm or 1.0°. Exclusion criteria included (1) patients who had a history of neurological and psychiatric disorders; (2) patients who abused alcohol, nicotine, or drugs; (3) patients who had a history of traumatic brain injury or brain disease; and (4) patients with metallic objects such as pacemakers implanted cannot undergo MRI. Healthy cases were additionally recruited for normal controls (NCs) with 42 participants.

All recruits completed laboratory tests, including hemoglobin, erythrocyte pressure product, albumin, urea nitrogen, creatinine, sodium, potassium, chloride, calcium, and phosphorus, 3 days before MRI imaging. All blood tests are performed in the same laboratory. Clinical information was collected from all recruits, including age, gender, history of hypertension, diabetes, hyperlipidemia, and duration of dialysis. All patients signed an informed consent form. The study was approved by the ethics committee of Liangxiang Hospital, Fangshan District, Beijing, China (ethics number 2016174).

### 2.2 Image acquisition

The MRI scans were performed using a 20-channel phased-array head coil on a Magnetom Skyra 3.0 T MRI scanner (Siemens, Germany). The parameters for all participants who underwent 3D T1-weighted structural image scanning were as follows: magnetization prepared rapid gradient echo (MP-RAGE) sequence with repetition time, TR = 2000 ms, inversion time TI = 880 ms, echo time TE = 2.01 ms, flip angle FA = 8^°^, matrix = 256 × 256, field of view FOV = 256 × 256 m2, total sagittal thickness = 208 mm, and thickness = 1 mm.

### 2.3 VBM analysis

The MRI data were analyzed using the CAT 12^1^ and a toolbox implemented in SPM 12.^2^ The preprocessing used default CAT 12 parameters, including the following steps: (i) segmentation of gray matter (GM), white matter (WM), and cerebrospinal fluid (CSF) and (ii) normalization to standard MNI space and modulating by scaling with the number of volume changes, followed by (iii) spatial smoothing with an 8 mm FWHM Gaussian kernel. Total intracranial volume (TIV) was also calculated as the covariances in the statistical analysis.

### 2.4 Assessment of cognitive function

This study used the Mini-Mental State Examination (MMSE) and the Montreal Cognitive Assessment (MoCA) to test cognitive function, which were widely used and recognized in clinical trials. The MMSE is one of the most widely used cognitive scales and the most commonly used tool in clinical trials to assess cognitive grading and screen for cognitive impairment. The MoCA complements the MMSE and provides a rapid screening of cognitive function. As a supplement to MISE, MOCA can quickly check the cognitive function of subjects and find patients with cognitive impairment missed by MMSE.

### 2.5 Statistical analysis

We used SPSS Statistics (version 26.0) to statistically analyze the clinical data and laboratory tests of the three groups. Age, dialysis duration, laboratory test indices, MMSE scores, and MoCA scores were expressed as mean ± standard deviation and compared using the one-way ANOVA (LSD method, satisfying independence, normality, and chi-square) or non-parametric tests (Kruskal–Wallis H-test, not satisfying normality, and chi-square). Gender, hypertension, diabetes, and hyperlipidemia were expressed as numbers and compared using a chi-square test. We compared the GM volume between the three groups using a two-sample *t*-test with age, gender, and TIV as covariates, which was corrected by using the Gaussian random field (GRF) method. Voxel *p* < 0.01 and cluster *p* < 0.05 after GRF correction were considered statistically significant. Correlation analysis was performed using R software (version 4.0.2), and *p* < 0.05 was considered statistically significant.

## 3 Results

### 3.1 Patient and laboratory tests

A total of 52 patients with CKD on dialysis, including 27 peritoneal dialysis patients with CKD (PD-CKD), 25 hemodialysis patients with CKD (HD-CKD), and 42 normal controls (NC), were recruited in our study. The mean age of the entire cohort was 56.82 ± 10.63 years. The mean duration of dialysis for all CKD patients was 63.423 ± 58.823 months. There were no significant differences in age and gender among the three groups except for patients with PD-CKD, who were significantly younger than patients with HD-CKD (*p* = 0.004). Patients with CKD had significantly higher rates of hypertension, hyperglycemia, and dyslipidemia than NCs. Patients with CKD showed significantly higher hemoglobin, erythrocyte pressure product, albumin, MMSE fraction, and MoCA fraction, as well as significantly lower urea nitrogen, creatinine, and phosphate levels than NCs. Patients with HD-CKD had a higher proportion of hyperglycemia and a shorter dialysis time (39.36 vs. 85.7 months, *p* = 0.004) than patients with PD-CKD. Detailed data are shown in Table 1.

**Table 1:** Demographic characteristics and laboratory tests of patients and healthy controls.

	NC (*n* = 42)	CKD (*n* = 52)		P	A	B	C
		PD-CKD (*n* = 27)	HD-CKD (*n* = 25)				
Demographics							
Age (years)	55.74 ± 9.85	54.63 ± 10.27	61.00 ± 11.47	0.064	0.655	0.051	0.004
Sex (Female)	18(42.86)	14(51.85)	10(40.00)	0.665	0.465	0.822	0.402
(Male)	24(57.14)	13(48.15)	15(60.00)				
High blood pressure	8(19.05)	25(92.59)	22(88.00)	<0.001	<0.001	<0.001	0.583
Diabetes	2(4.76)	12(44.44)	20(80.00)	<0.001	<0.001	<0.001	0.008
High blood cholesterol	3(7.14)	18(66.67)	18(72.00)	<0.001	<0.001	<0.001	0.684
Duration of dialysis (months)	–	85.70 ± 69.13	39.36 ± 31.85	0.004	–	–	0.004
Laboratory tests							
Hemoglobin (g/dL)	134.83 ± 15.94	107.78 ± 24.17	105.04 ± 20.28	<0.001	<0.001	0.001	0.012
Erythrocyte pressure (%)	40.41 ± 4.12	32.96 ± 7.37	32.76 ± 6.55	<0.001	<0.001	<0.001	0.078
Albumin (g/dL)	38.40 ± 3.22	34.22 ± 5.56	33.76 ± 4.49	<0.001	<0.001	<0.001	0.626
Urea nitrogen(mg/dL)	5.04 ± 1.51	21.20 ± 9.79	16.42 ± 6.19	<0.001	<0.001	<0.001	1.000
Creatinine (mg/dL)	65.76 ± 16.92	1052.07 ± 433.10	704.48 ± 243.23	<0.001	<0.001	<0.001	1.000
Sodium (mmol/L)	139.48 ± 2.03	138.85 ± 3.55	139.88 ± 2.83	0.397	0.356	0.500	0.256
Potassium (mmol/L)	4.10 ± 0.48	4.31 ± 0.74	4.43 ± 0.84	0.140	0.156	0.049	0.607
Chloride (mmol/L)	98.55 ± 15.71	95.93 ± 5.17	98.92 ± 4.33	0.550	0.406	0.909	0.029
Calcium (mg/dL)	2.33 ± 0.12	2.29 ± 0.30	2.25 ± 0.16	0.333	0.475	0.038	0.589
Phosphorus(mg/dL)	1.10 ± 0.28	1.68 ± 0.51	1.77 ± 0.77	<0.001	<0.001	<0.001	0.605
MMSE Score	28.57 ± 1.25	24.22 ± 2.87	23.72 ± 3.47	<0.001	<0.001	<0.001	0.571
MoCA Scores	27.74 ± 1.64	24.11 ± 2.12	24.48 ± 2.93	<0.001	<0.001	<0.001	0.603

P: comparison between the NC and CKD groups (NC vs. CKD). A: comparison between the NC and PD-CKD groups (NC vs. PD-CKD). B: comparison between the NC and HD-CKD groups (NC vs. HD-CKD). C: comparison between the PD-CKD and HD-CKD groups (PD-CKD vs. HD-CKD).

### 3.2 Analysis of cerebral gray matter volume changes in peritoneal dialysis and hemodialysis groups

**Figure 1:**
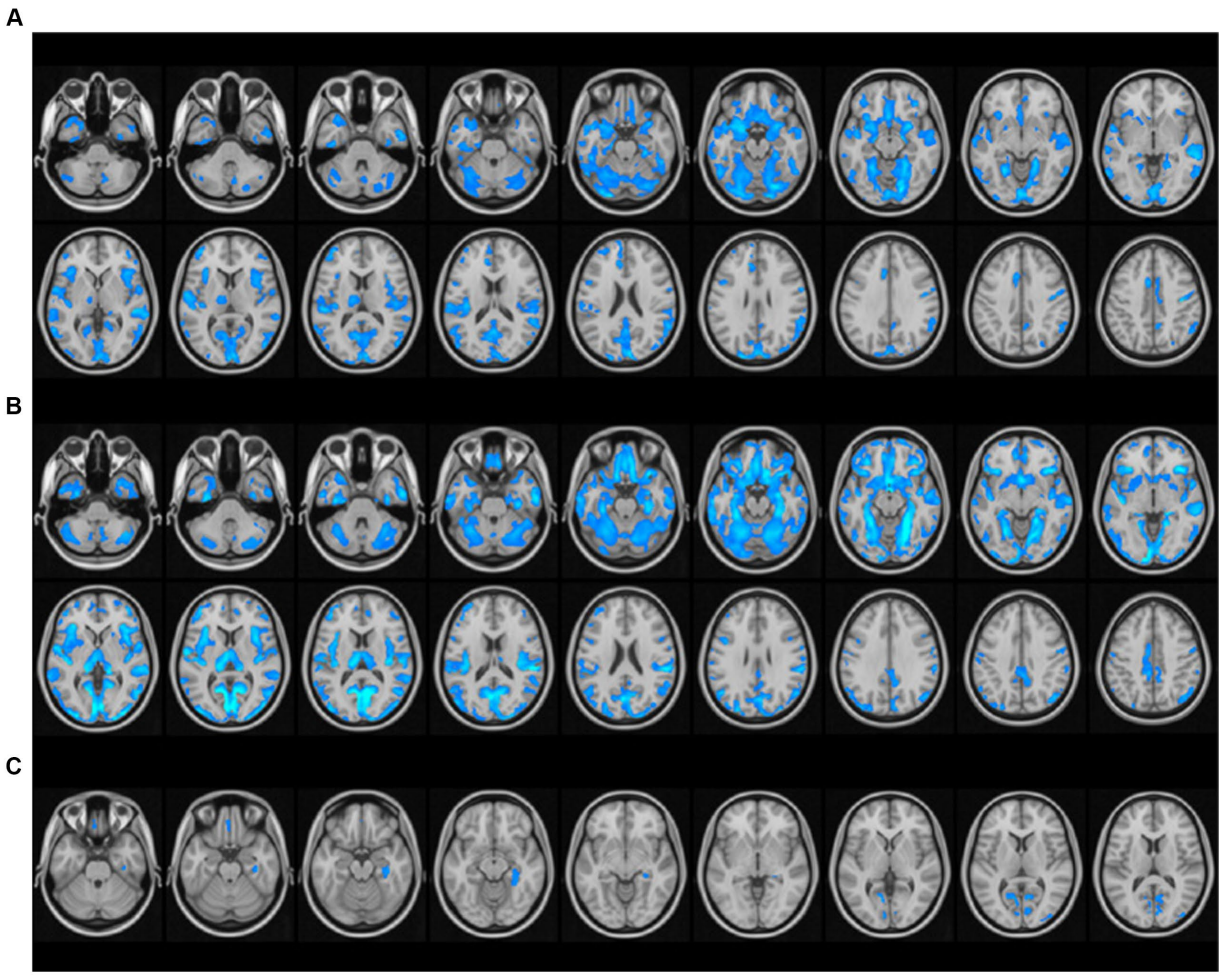
Differential analysis of brain gray matter volume among the peritoneal dialysis group, hemodialysis group, and healthy control group. **(A)** PD-CKD compared to NC, regions with significantly lower GMV; **(B)** HD-CKD compared to NC, areas with significantly lower GMV; **(C)** Areas of HD-CKD with significantly lower GMV compared to PD-CKD.

Compared with the control group, patients with PD-CKD and HD-CKD had significantly reduced gray matter volumes in the bilateral middle temporal gyrus, bilateral periseptal laminae, bilateral superior temporal gyrus, bilateral cerebellar area 6, bilateral gyrus, bilateral cerebellar Crus1 area, left inferior temporal gyrus, bilateral syrinx gyrus, and bilateral insula areas. Moreover, patients with PD-CKD had reduced left cuneate lobe volumes, and HD-CKD patients had reduced gray matter volumes in the right inferior temporal gyrus, bilateral middle occipital gyri, bilateral cerebellar area 8, bilateral inferior frontal gyrus orbits, bilateral thalamus, bilateral rectus gyrus, left medial and para-cingulate gyrus, right parahippocampal gyrus, bilateral cerebellar area Crus2, bilateral central sulcus cap, right angular gyrus, and right hippocampal area than NCs. Patients with HD-CKD had reduced gray matter volumes in the bilateral periapical lamina of the talar fissure, right parahippocampal gyrus, right cingulate gyrus, right hippocampus, left rectus gyrus, and right middle occipital gyrus regions than patients with PD-CKD (Table 2 and Figure 1).

**Table 2:** Changes in gray matter volume between the three groups.

PD-CKD vs. NC		HD-CKD vs. NC		HD-CKD vs. PD	
Num.	Region	Num.	Region	Num.	Region
2,680	Temporal_Mid_L	3,855	Temporal_Mid_R	491	Calcarine_R
2,670	Calcarine_L	3,508	Temporal_Mid_L	406	Calcarine_L
2,628	Temporal_Mid_R	3,465	Fusiform_L	311	ParaHippocampal_R
2,221	Temporal_Sup_R	3,379	Temporal_Inf_R	200	Fusiform_R
2099	Cerebelum_6_L	2,850	Cerebelum_6_R	151	Hippocampus_R
1967	Lingual_R	2,709	Calcarine_L	146	Rectus_L
1958	Temporal_Sup_L	2,564	Fusiform_R	119	Occipital_Mid_R
1750	Cerebelum_Crus1_L	2,474	Insula_R		
1747	Temporal_Inf_L	2,420	Lingual_R		
1738	Fusiform_L	2,401	Cerebelum_6_L		
1717	Cerebelum_6_R	2,396	Temporal_Inf_L		
1,469	Cerebelum_Crus1_R	2,327	Occipital_Mid_L		
1,461	Fusiform_R	2,268	Lingual_L		
1,460	Lingual_L	2,128	Cerebelum_8_R		
1,438	Calcarine_R	2,124	Temporal_Sup_R		
1,304	Cuneus_L	1987	Insula_L		
1,270	Insula_R	1975	Frontal_Inf_Orb_L		
1,095	Insula_L	1952	Cerebelum_Crus1_R		
		1949	Calcarine_R		
		1,662	Temporal_Sup_L		
		1,578	Frontal_Inf_Orb_R		
		1,454	Occipital_Mid_R		
		1,452	Cerebelum_Crus1_L		
		1,406	Thalamus_L		
		1,349	Rectus_L		
		1,298	Cerebelum_8_L		
		1,276	Cingulum_Mid_L		
		1,256	ParaHippocampal_R		
		1,218	Rectus_R		
		1,196	Rolandic_Oper_R		
		1,102	Thalamus_R		
		1,075	Cerebelum_Crus2_L		
		1,048	Rolandic_Oper_L		
		1,045	Cerebelum_Crus2_R		
		1,011	Angular_R		
		1,008	Hippocampus_R		

For PD-CKD vs. NC and HD-CKD vs. NC, regions with voxels > 1,000 were listed based on the AAL atlas. For HD-CKD vs. PD-CKD, regions with voxels > 100 were listed based on AAL (Num: number of voxels). AAL: Anatomical Automatic Labeling.

### 3.3 Correlative analysis of gray matter volume change and cognitive score

The correlation results showed that both MMSE and MoCA scores were positively correlated with the amount of gray matter volume changes in bilateral cerebellar area 6, bilateral amygdala, bilateral perirhinal cortex, bilateral hippocampus, bilateral lingual gyrus, left superior temporal gyrus, left polar rectus, left olfactory bulb, right cuneus, and right central sulcus (See Table 3).

**Table 3:** Correlation of gray matter volume change and cognitive score.

Cluster	MMSE			MoCA		
	Correlation coefficients	Num	Region	Correlation coefficients	Num.	Region
1	0.544	954	Temporal_Sup_L	0.559	1700	Cerebelum_6_L
		608	Insula_L		1,574	Cerebelum_6_R
		601	Temporal_Pole_Sup_L		1,357	Lingual_R
		411	Rectus_R		1,313	Calcarine_L
		403	Rolandic_Oper_L		1,084	Lingual_L
		386	Olfactory_L		1,028	Fusiform_L
		338	Hippocampus_L		897	Cerebelum_Crus1_R
		306	Temporal_Pole_Mid_L		879	Calcarine_R
		277	Rectus_L		878	Fusiform_R
		268	Frontal_Inf_Orb_L		795	Cerebelum_Crus1_L
		265	Amygdala_L		378	Cerebelum_4_5_R
		249	Amygdala_R		241	Cuneus_L
		245	Olfactory_R			
		208	Hippocampus_R			
2	0.535	1,404	Lingual_R	0.475	716	Temporal_Sup_L
		1,324	Calcarine_L		489	Temporal_Pole_Sup_L
		1,087	Calcarine_R		381	Rectus_L
		1,029	Fusiform_L		368	Olfactory_L
		932	Lingual_L		345	Hippocampus_L
		914	Fusiform_R		271	Rectus_R
		698	Cerebelum_6_R		265	Amygdala_L
		606	Cerebelum_6_L			
		295	Cuneus_R			
		237	Cuneus_L			
3	0.417	737	Temporal_Mid_R	0.455	272	Hippocampus_R
		429	Temporal_Sup_R		228	Amygdala_R
4	0.466	295	Rolandic_Oper_R	0.422	622	Insula_R
		255	Temporal_Sup_R		228	Rolandic_Oper_R

**Figure 2:**
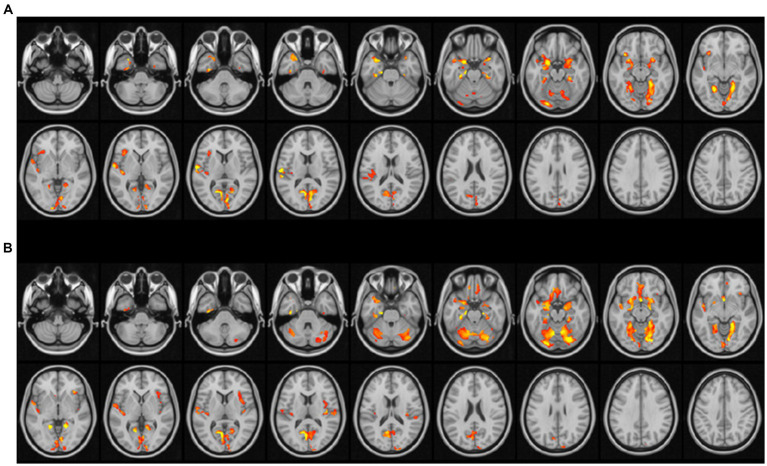
Correlation of gray matter volume change and cognitive score. **(A)** Red indicates brain areas significantly positively correlated with MMSE; **(B)** red indicates brain areas significantly positively correlated with MOCA (*p* < 0.01 for waxel and *p* < 0.05 for cluster after GFR correction with age, sex, and TIV as covariates).

Moreover, MMSE scores were positively correlated with the amount of gray matter volume change in the right superior temporal gyrus, left insula, right rectus gyrus, left central sulcus cap, left middle temporal gyrus, left inferior frontal gyrus orbit, right olfactory cortex, bilateral perirhinal fissure cortex, bilateral syrinx, left cuneus, and right middle temporal gyrus regions. MoCA scores were positively correlated with the amount of gray matter volume change in the bilateral syrinx, right cerebellar Crus1 region, right cerebellar regions 4 and 5, right rectus gyrus, and right insula regions. All of the above correlation coefficients ranged from 0.4 to 0.6 (See Figure 2).

## 4 Discussion

A growing number of studies have shown an increased risk of cognitive impairment in patients with CKD, and dialysis further improves risk (Penton et al., 2020). Patients with ESRD can exhibit significant brain atrophy and cognitive impairment after dialysis (Zhang et al., 2022), which were consistent with the results of our study. However, the mechanisms of cognitive impairment in patients with CKD have not been elucidated. Recent studies suggest that cognitive impairment and dementia in CKD patients may lead to disorders of the renal-brain axis (Tsuruya and Yoshida, 2018; de Miranda et al., 2022). Both brain and renal tissues are low blood flow resistance organs with high blood flow requirements and are susceptible to blood pressure. Previous studies have reported that the hemodynamic characteristics of dialysis patients are special and unstable, which can easily lead to vascular endothelial injury. This endothelial injury can cause microcirculatory damage, leading to neurovascular unit damage in the cerebral cortical system, resulting in cognitive decline in patients. Moreover, factors such as toxin sequelae, anemia, lipid metabolism disorders, and poor nutritional status are also closely related to cognitive function in patients with CKD (Zhao et al., 2022).

Our results showed that different dialysis modalities could cause damage to specific areas of the brain, but there was variability in the damage caused by different modalities. Dialysis-induced blood pressure fluctuations are associated with brain injury (Mizumasa et al., 2004; McIntyre, 2010). Hemodialysis shows a greater impact on hemodynamic stability than peritoneal dialysis (Daugirdas, 2001; Zhang et al., 2014; Yang et al., 2024). Hemodialysis is considered to have more unstable hemodynamic changes due to the use of anticoagulants. It seems that hemodialysis should show a higher incidence of brain injury than peritoneal dialysis. However, the prevalence of cognitive impairment in peritoneal dialysis patients remains high (Kalirao et al., 2011; Li et al., 2017), which illustrates the mechanistic complexity and non-singularity of dialysis-induced brain injury. Therefore, further research is needed to demonstrate the specific mechanisms affecting changes in brain damage in patients with CKD on different dialysis modalities.

The duration of dialysis may also affect the cognitive function of the brain. Our study showed that the mean duration of peritoneal dialysis was 39.36 months and 85.7 months. The longer duration of dialysis in hemodialysis compared to peritoneal dialysis may lead to longer and irreversible changes in cerebral hemodynamics, resulting in more severe brain damage in hemodialysis patients. It has been found that cerebral microbleeds caused by routine use of anticoagulants during hemodialysis may also induce cognitive impairment (Watanabe, 2007).

MMSE and MoCA scores can assess cognitive function, but subjective factors affect the accuracy of assessment results. Our study found that the MMSE and MoCA scores were positively correlated with brain gray matter volume changes. VBM is a non-invasive technique that covers the whole brain and can be used to detect changes regularly during dialysis in patients. Consequently, the integration of VBM with the MMSE and the MoCA enhances the precision of cognitive impairment assessment. This amalgamation facilitates the dynamic monitoring of cerebral damage in patients undergoing dialysis, enabling the early detection of latent brain injuries and the initiation of timely intervention treatments. Such an approach not only prognosticates an improvement in patient outcomes but also elevates their quality of life. This synergy underscores the importance of a multidimensional assessment framework in the early stages of cognitive decline, suggesting that a comprehensive evaluation surpasses the capabilities of a singular diagnostic tool. Future studies should continue to explore integrating neuroimaging techniques with cognitive screening tools to refine diagnostic accuracy and treatment efficacy, thereby bolstering patient care and prognostic outcomes. However, due to the limited number of patients in this study, further verification and improvement on a larger dataset are needed. This is also the next step of our research.

## 5 Conclusion

We found that different dialysis modalities cause different damage to specific areas of the brain through VBM. Moreover, VBM can be combined with the MMSE and MoCA to detect structural brain changes and cognitive impairment in patients with different dialysis modalities. VBM deserves further exploration of its comprehensive application in the field of neurological function.

## Data availability statement

The raw data supporting the conclusions of this article will be made available by the authors, without undue reservation.

## Author contributions

FX: Methodology, Investigation, Writing – original draft. LZ: Writing – review & editing, Investigation. YaL: Methodology, Data curation, Writing – review & editing. CZ: Writing – original draft, Validation. YiL: Writing – review & editing, Methodology. HY: Writing – original draft, Conceptualization. XL: Writing – review & editing, Visualization. CW: Writing – review & editing, Investigation. XY: Writing – original draft, Methodology, Formal analysis. XG: Writing – review & editing, Validation, Supervision.

## Footnotes

^1^ http://www.neuro.uni-jena.de/software/

^2^ http://www.fil.ion.ucl.ac.uk/spm/software/spm12

## References

[ref9] Bikbov B. Purcell C. A. Levey A. S. Smith M. Abdoli A. Abebe M.. (2020). Global, regional, and national burden of chronic kidney disease, 1990–2017: a systematic analysis for the global burden of disease study 2017. Lancet 395, 709–733. doi: 10.1016/S0140-6736(20)30045-3, PMID: 32061315 PMC7049905

[ref1] Daugirdas J. T. (2001). Pathophysiology of dialysis hypotension: an update. Am. J. Kidney Dis. 38, S11–S17. doi: 10.1053/ajkd.2001.28090, PMID: 11602456

[ref2] de Miranda A. S. Macedo D. S. Rocha N. P. Teixeira A. L. (2022). Targeting the renin-angiotensin system (RAS) for neuropsychiatric disorders. Curr. Neuropharmacol. 22:107. doi: 10.2174/1570159X20666220927093815 PMC10716884 36173067

[ref3] Foreman K. J. Marquez N. Dolgert A. Fukutaki K. Fullman N. McGaughey M.. (2018). Forecasting life expectancy, years of life lost, and all-cause and cause-specific mortality for 250 causes of death: reference and alternative scenarios for 2016-40 for 195 countries and territories. Lancet 392, 2052–2090. doi: 10.1016/S0140-6736(18)31694-5, PMID: 30340847 PMC6227505

[ref4] Gamage I. Dhar A. Tregaskis P. Wilson S. (2022). Frequency and risk factors for cognitive dysfunction in peritoneal dialysis patients. Nephrology 27, 945–952. doi: 10.1111/nep.14117, PMID: 36190395

[ref5] Hiramatsu T. Okumura S. Asano Y. Mabuchi M. Iguchi D. Furuta S. (2020). Quality of life and emotional distress in peritoneal Dialysis and hemodialysis patients. Ther. Apher. Dial. 24, 366–372. doi: 10.1111/1744-9987.13450, PMID: 31671240

[ref6] Jia X. Wang Z. Huang F. Su C. du W. Jiang H.. (2021). A comparison of the Mini-mental state examination (MMSE) with the Montreal cognitive assessment (MoCA) for mild cognitive impairment screening in Chinese middle-aged and older population: a cross-sectional study. BMC Psychiatry 21:485. doi: 10.1186/s12888-021-03495-6, PMID: 34607584 PMC8489046

[ref7] Kalantar-Zadeh K. Jafar T. H. Nitsch D. Neuen B. L. Perkovic V. (2021). Chronic kidney disease. Lancet 398, 786–802. doi: 10.1016/S0140-6736(21)00519-5 34175022

[ref8] Kalirao P. Pederson S. Foley R. N. Kolste A. Tupper D. Zaun D.. (2011). Cognitive impairment in peritoneal dialysis patients. Am. J. Kidney Dis. 57, 612–620. doi: 10.1053/j.ajkd.2010.11.026, PMID: 21295896 PMC3121243

[ref10] Li W. K. Chen Y. C. Xu X. W. Wang X. Gao X. (2022). Human-guided functional connectivity network estimation for chronic tinnitus identification: a modularity view. IEEE J. Biomed. Health Inform. 26, 4849–4858. doi: 10.1109/JBHI.2022.3190277, PMID: 35830394

[ref11] Li W. Qiao L. Zhang L. Wang Z. Shen D. (2019a). Functional brain network estimation with time series self-scrubbing. IEEE J. Biomed. Health Inform. 23, 2494–2504. doi: 10.1109/JBHI.2019.2893880, PMID: 30668484 PMC6904893

[ref12] Li W. Wang Z. Zhang L. Qiao L. Shen D. (2017). Remodeling Pearson's correlation for functional brain network estimation and autism Spectrum disorder identification. Front. Neuroinform. 11:55. doi: 10.3389/fninf.2017.00055, PMID: 28912708 PMC5583214

[ref13] Li W. Xu X. Wang Z. Peng L. Wang P. Gao X. (2021). Multiple connection pattern combination from single-mode data for mild cognitive impairment identification. Front. Cell Dev. Biol. 9:782727. doi: 10.3389/fcell.2021.782727, PMID: 34881247 PMC8645991

[ref14] Li W. Zhang L. Qiao L. Shen D. (2019b). Toward a better estimation of functional brain network for mild cognitive impairment identification: a transfer learning view. IEEE J. Biomed. Health Inform. 24, 1160–1168. doi: 10.1109/JBHI.2019.2934230 31403449 PMC7285887

[ref15] McIntyre C. W. (2010). Recurrent circulatory stress: the dark side of dialysis. Semin. Dial. 23, 449–451. doi: 10.1111/j.1525-139X.2010.00782.x, PMID: 21039872

[ref16] Mizumasa T. Hirakata H. Yoshimitsu T. Hirakata E. Kubo M. Kashiwagi M.. (2004). Dialysis-related hypotension as a cause of progressive frontal lobe atrophy in chronic hemodialysis patients: a 3-year prospective study. Nephron Clin. Pract. 97, c23–c30. doi: 10.1159/000077592, PMID: 15153764

[ref17] Penton A. A. Lau H. Babikian V. L. Shulman J. Cervantes-Arslanian A. Gangadhara S.. (2020). Chronic kidney disease as risk factor for enlarged perivascular spaces in patients with stroke and relation to racial group. Stroke 51, 3348–3351. doi: 10.1161/STROKEAHA.119.028688, PMID: 33019895 PMC7606816

[ref18] Tsuruya K. Yoshida H. (2018). Brain atrophy and cognitive impairment in chronic kidney disease. Contrib. Nephrol. 196, 27–36. doi: 10.1159/000485694, PMID: 30041201

[ref19] Watanabe A. (2007). Cerebral microbleeds and intracerebral hemorrhages in patients on maintenance hemodialysis. J. Stroke Cerebrovasc. Dis. 16, 30–33. doi: 10.1016/j.jstrokecerebrovasdis.2006.08.004, PMID: 17689389

[ref20] Yang J. Xu X. Sun M. Ruan Y. Sun C. Li W.. (2024). Towards an accurate autism spectrum disorder diagnosis: multiple connectome views from fMRI data. Cereb. Cortex 34:477. doi: 10.1093/cercor/bhad477, PMID: 38100334

[ref21] Zacková L. Jáni M. Brázdil M. Nikolova Y. S. Marečková K. (2021). Cognitive impairment and depression: Meta-analysis of structural magnetic resonance imaging studies. Neuroimage Clin 32:102830. doi: 10.1016/j.nicl.2021.102830, PMID: 34560530 PMC8473769

[ref22] Zhang X. Liu C. Nepal S. Yang C. Dou W. Chen J. (2014). A hybrid approach for scalable sub-tree anonymization over big data using MapReduce on cloud. J. Comput. Syst. Sci. 80, 1008–1020. doi: 10.1016/j.jcss.2014.02.007

[ref23] Zhang C. Yu H. Cai Y. Wu N. Liang S. Zhang C.. (2022). Diffusion tensor imaging of the brain white matter microstructure in patients with chronic kidney disease and its correlation with cognition. Front. Neurol. 13:1086772. doi: 10.3389/fneur.2022.1086772, PMID: 36588888 PMC9798235

[ref24] Zhao Y. Song P. Zhu C. Zhang L. Chen X. Zhang H.. (2022). Relationship between physical performance and mild cognitive impairment in elderly hemodialysis patients is modified by the presence of diabetes: a multicenter cross-sectional study. Front Endocrinol 13:897728. doi: 10.3389/fendo.2022.897728, PMID: 36157461 PMC9501887

